# The interaction between Sertoli cells and luekemia inhibitory factor on the propagation and differentiation of spermatogonial stem cells in vitro

**Published:** 2015-11

**Authors:** Tayebeh Rastegar, Mehryar Habibi Roudkenar, Soraya Parvari, Maryam Baazm

**Affiliations:** 1 *Department of Anatomy, School of Medicine, Tehran University of Medical Sciences, Tehran, Iran.*; 2 *Blood Transfusion Research Center, High Institute for Research and Education in Transfusion Medicine, Tehran, Iran.*; 3 *Department of Anatomy, School of Medicine, Alborz University of Medical Sciences, Karaj, Iran.*; 4 *Department of Anatomy, School of Medicine, Arak University of Medical Sciences, Arak, Iran.*

**Keywords:** *Spermatogonial stem cells*, *Sertoli cell*, *Proliferation*, *Differentiation*, *Co-culture*, *Luekemia inhibitory factor*

## Abstract

**Background:**

Sertoli cells play a pivotal role in creating microenvironments essential for spermatogonial stem cells (SSCs) self-renewal and commitment to differentiation. Maintenance of SSCs and or induction of in vitro spermiogenesis may provide a therapeutic strategy to treat male infertility.

**Objective:**

This study investigated the role of luekemia inhibitory factor (LIF) on the propagation of SSCs and both functions of Sertoli cells on the proliferation and differentiation of these cells.

**Materials and Methods:**

SSCs were sorted from the testes of adult male mice by magnetic activated cell sorting and thymus cell antigen 1 antibody. On the other hand, isolated Sertoli cells were enriched using lectin coated plates. SSCs were cultured on Sertoli cells for 7 days in the absence or presence of LIF. The effects of these conditions were evaluated by microscopy and expression of meiotic and post meiotic transcripts by reverse transcriptase polymerase chain reaction.

**Results:**

Our data showed that SSCs co-cultured with Sertoli cells in the presence of LIF formed colonies on top of the Sertoli cells. These colonies had alkaline phosphatesase activity and expressed SSCs specific genes. SSCs were enjoyed limited development after the mere removal of LIF, and exhibiting expression of meiotic and postmeiotic transcript and loss of SSCs specific gene expression (p< 0.05).

**Conclusion:**

Our findings represent co-culture of SSCs with Sertoli cells provides conditions that may allow efficient proliferation and differentiation of SSCs for male infertility treatment**.**

## Introduction

The spermatogonial stem cells (SSCs) are the foundation of spermatogenesis. They can self-renew and produce a large number of differentiated germ cells in vivo (1). Sometimes, male fertility is related to spermatogenic arrest. In these cases, interruption of germ cell differentiation leads to azoospermia or oligozoospermia (2). In vitro maturation of germ cells and promoting their differentiation may be helpful for these patients (3). Because of the rare number of SSCs in the testis, proliferation of these cells is required. It has been previously demonstrated that the incubation of mouse SSCs in presence of feeder cells or growth factors enables their long-term proliferation in vitro (4). In this study, it was attempted to use Sertoli cells as feeder layer not only for proliferation, for differentiation of SSCs.

Sertoli cells are the somatic cells of the testis and play pivotal role in testis formation in the embryo and spermatogenesis in the adult males also, provide a structural and nutritional support for germ cells (1, 5). There is a specialized interaction between Sertoli cells and germ cells (6, 7).

The molecular control of germ cell fate is precisely regulated by intrinsic gene expression in the SSCs and extrinsic factors from testis niche (5, 8, 9). Sertoli cells thigh junctions, the main structural component of the seminiferous tubule, provide a restricted tissue environment for SSCs (10). The paracerine regulations and cell-cell adhesion through gap junctions facilitate interactions between SSCs and Sertoli cells. Sertoli cells produce different growth factors involving in proliferation and differentiation of SSCs (11).

The important factor that is necessary for SSCs proliferation and maintenance in vitro and in vivo is Glial derived neurotrophic factor (GDNF) (12). GDNF is a Sertoli-cell-derived factor that controls the balance between self-renewal and differentiation of the spermatogonial stem cells (13). Basic fibroblast growth factor and epidermal growth factor (EGF) are the other necessary factors, secreted by Sertoli cells, for SSCs self-renewal (14).

In addition, Sertoli cells express follicle stimulating factor (FSH) and testosterone receptors (5). Both of these hormones are necessary for spermeiogenesis. The high concentration of FSH could stimulate germ cell meiosis in vitro (15). On the other hand, FSH could regulate SSCs proliferation by GDNF/FSH pathway (16). Therefore, Sertoli cells regulate SSCs proliferation and differentiation by different mechanisms. LIF is the other growth factor that its role in survival of embryonic stem cells has been identified (17). In testis, LIF is secreted by peritubular cells of seminiferous tubules and acts via its receptors. SSCs express LIF receptors and are able to bind to LIF, whereas these receptors are absent on the meiotic cells (18). Therefore, in this study, this cytokine was used for proliferation of SSCs.

Some data demonstrate an improvement in culture system over time through changes in the culture medium composition and in the germ cell microenvironment (4). Today, spermatogonial and testicular somatic cells culture has become important and innovative tools for applied research in reproductive biology (19). Development of in vitro differentiation assays would help in introducing culturing condition to induce spermatogenesis process in SSCs (20).

In this study, it was co-cultured adult SSCs with primary culture of Sertoli cells to replicate conditions somewhat similar to the natural niche applicable in clinical approach. By using this condition, it was investigated the potential role of Sertoli cells for increasing the number of SSCs and following efficient differentiation of SSCs to spermatid-like cells.

## Materials and methods

**Experimental animals**

In this experimental study, four to six week-old national medical research institute (NMRI) male mice were maintained under standard conditions with free access to food and water. The ethics committee of Arak University of Medical Sciences approved the animal experiments (91-133-5), in accordance with University guidelines.

**SSC enrichment**

For obtaining testicular cells, a modified method published by Guan was used (21). Briefly, decapsulated testis was cut into small pieces and seminiferous tubules were transferred to collagenase type I (1 mg/ml) (Sigma, Germany), Deoxyribonuclease I (10 μg/ml) (DNase; Sigma, Germany) solution. Cells were incubated at 37˚C in a CO_2_ incubator and dispersed by pipetting every 2–5 min until the tubules separated after about 20 min. Cells were washed twice with 10 ml of phosphate buffered saline (PBS; Sigma, Germany) as reported previously. The dissociated testis cell suspension was overlaid on 30% (v/v) Percoll (Sigma, Germany) prepared in PBS containing 1% fetal bovine serum (FBS; Gibco, USA) and centrifuged at 600×g for 8 min at 4˚C. For magnetic-activated cell sorting (MACS), Miltenyi Biotec protocol was used. Sedimented cells (bottom fraction) of the Percoll gradient (3–8×10⁶ cells in 90 μl of PBS) were incubated with 10 μl of thymus cell antigen 1 (Thy-1) antibody (30-H12; Miltenyi Biotec, Germany) microbeads for 20 min at 4˚C. After rinsing with PBS containing 0.5% bovine serum albumin (BSA; Sigma, Germany), Thy-1^+^ cells were selected by passing them through a large separating column (Miltenyi Biotec, Germany) that was placed in a magnetic field.

**Flow cytometry**

Flow cytometric analysis was performed on populations of testis cells before and after enrichment. Briefly, 10^6^ cells were suspended in 0.1 ml of PBS/1% FBS and 10μl: fluorescence isothiocyanate conjugated hamster anti-rat β1-integrin (CD29) (R&D, USA). Antibody was added to the cells for 20 min at 4^°^C. The cells were washed twice in 1 ml of PBS/FBS. Then, 0.1 ml of PBS/1% FBS and 10μl phycoerythrin (PE) conjugated rat anti-human a6-integrin (CD49f) (R&D, USA) was added to the cells for 20 min at 4^°^C. The cells were washed twice with 1 ml of PBS/FBS. Control cells were not treated with antibody (22). 

**Sertoli cells isolation and culture**

Adult Sertoli cells were extracted from the testis of adult mice as previously described (23). Briefly, culture dishes were coated with 5 μg/ml of lectin Datura stramonium agglutinin (DSA; Sigma, Germany) in PBS at 37^°^C for 1 h. 

Then, the coated plastic dishes were washed three times with 0.5% BSA. The mixed population of the cells obtained by enzymatic digestion was placed on the lectin-coated dishes and incubated for 1 h at 32^°^C in a humidified atmosphere of 5% CO_2_. After incubation, the non-adhering cells were collected and washed twice with Dulbecco's modified eagle (DMEM; Gibco, USA). Then, DMEM and 10% FBS were added. After 7 days the Sertoli cells formed a confluent layer.

**Immunocytochemistry for characterization of adult Sertoli cells**

Vimentin was detected in cultured Sertoli cells by immunocytochemistry. After fixation with 4% paraformaldehyde (Sigma, Germany), permeabilization by 0.4% Triton X100 (Sigma, Germany) and blocking with 10% goat serum (Sigma, Germany), the cells were incubated for 2 h at 37^°^C with mouse monoclonal anti-Vimentin antibody diluted 1:100 (Sigma, Germany). 

After washing with PBS, the secondary antibody, goat anti-mouse labeled with fluorescence isothiocyanate (Sigma, Germany) diluted 1:100, was applied for 3 h. Control cells were treated under similar conditions, while instead of the primary antibody, horse serum was added. Nuclei were stained (5μg/ml Propidium iodide; PI; Sigma, Germany) (24).

**SSCs proliferation and differentiation**

Enriched SSCs were cultured (6–10 ×10⁴ cells/cm^2^) in Minimum essential medium alpha (MEMα; Gibco, USA) containing 10% FBS, 1 x non-essential amino acids (Gibco, USA), 0.1 mM 2-mercaptoethanol )Sigma, Germany), 103U/ml LIF (B&D, USA), 0.4mM Pyruvate (Sigma, Germany), 1x Glutamine (Gibco, USA), 100u/ml Penicillin and 100 μg/ml Streptomycin (Sigma, Germany), on Sertoli cells or gelatin (Sigma, Germany) coated dishes for 7 days. In another groups the cells were cultured in a MEMα medium containing the above-mentioned without LIF, co-culturing with gelatin or Sertoli cells. The culture medium was changed every 3 day. All culture media were maintained at 32˚C in an atmosphere humidified with 5% CO_2_ (21). SSCs colonies were then stained with Alkaline Phosphatase (25). The SSCs number was determined with a hemacytometer. Trypan blue (0.4%) (Sigma, Germany) exclusion assays were used to determine the percentage of the surviving cells following the isolation and culture (26).

**Reverse transcriptase polymerase chain reaction**

After 7 days, the expression of specific genes such as Stimulated by retinoic acid gene 8 (Stra8) and Detected in azoospermia- like (Dazl) expressed in SSCs, H2A, Synaptonemal complex protein 3 (Scp3), and TH2B expressed in spermatocytes, and Ubiquitin-activated enzyme (Ube1y), Transition protein 1(TP1), TP2 and Protamine 1 (PRM1) expressed in spermatids were studied by RT-PCR. Table I gives primer sequences. Total RNA was extracted by Trizol reagent (Invitrogen, CA) according to the manufacturer's protocol. 

RT-PCR using a Complementary DNA (cDNA) synthesis kit (Bioneer, South Korea) using 1 μl of total RNA was performed according to the manufacturer's protocol. RT-PCR was performed using Tag DNA polymerase (Cinagene, Iran) in a Gene Amp PCR system 9600 (PerkinElmer Life and Analytical Sciences, MA). After an initial denaturation at 94^°^C for 5 min, cDNA was subjected to 33 cycles of PCR. 

Testicular cells from adult testis were used as positive control and liver cells were used as negative control. To normalize RT-PCR samples, expression of β-actin was examined. The PCR products on 2% agarose gels was observed, and the intensity of bands by UVI doc gel documentation system was assayed (Cambridge, CB4 1QB-UK).

**Statistical analysis**

The results were expressed as Mean ± standard deviation (SD). The statistical significance between the mean values was determined by one-way analysis of variance (ANOVA), Tukey and Duncan post-test. A level of p≤0.05 was considered as the level of significance.

## Results

**SSCs enrichment**

**Figure 1:**
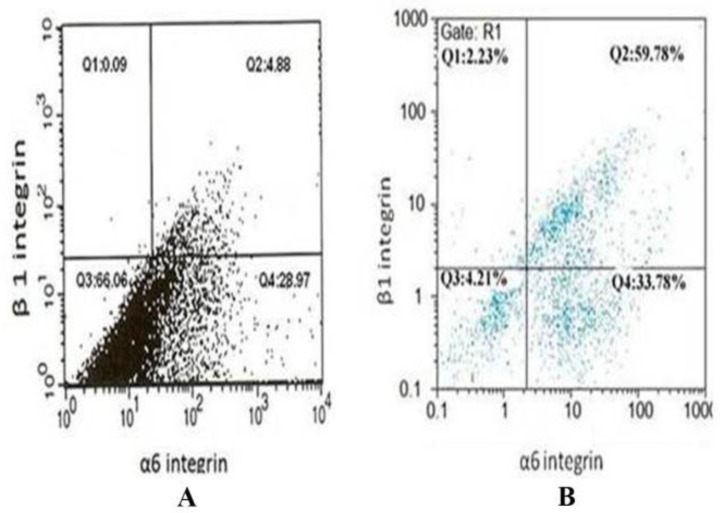
Flow cytometric analysis for detection of α6 and β1-integrin in SSCs. While there are low cells with expression of β1 and α6-integrins before MACS (A), there are considerable cells with positive β1 and α6-integrins following MACS purification (B).

In the current study, SSCs were isolated using MACS method and anti-Thy-1 antibody. The purity of the isolated cells was determined by flow cytometery using α6 and β1-integrin antibodies. The flow cytometric analysis revealed enriched Thy-1 purified cells, 67.2 ± 2.2% (n=6) expressed β1 integrin, 37.7 ± 1.8% (n=6) expressed α6 integrin, and 30.8 ± 1.9% (n=6) of Thy-1-enriched cells expressed both integrins (Figure 1), consistent with previous data (51.3 ± 11.9) for β1 integrin (22).

**Sertoli cell isolation and characterization**

**Figure 2:**
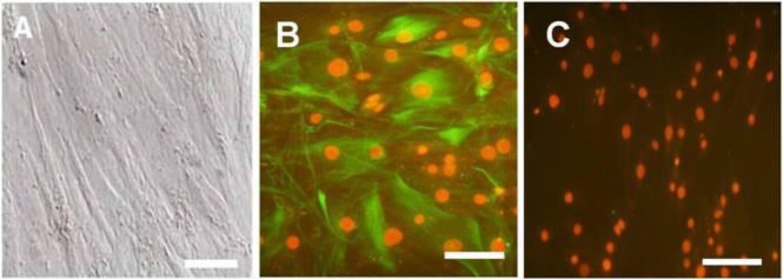
Microscopy morphology of Sertoli cells derived from 6-8 week-old male mice. Sertoli cells were isolated by lectin DSA and characterized by immunocytochemistry. Monolayer Sertoli cells started to flatten and spread out following one day plating on lectin DSA (x 200) (A). Sertoli cells were positive for Vimentin in cytoplasm (green color) and nuclei were stained with propidium iodide (x 200) (B) (orange color), Negative control (x 200) (C). Bar 50µm

Cells cultured on lectin DSA-coated plates were initially rounded and adhered firmly to the bottom of the dish. After 1 day, the cells started to flatten; they gradually spread and took on an epithelioid appearance (Figure 2A). The presence of Vimentin protein on Sertoli cells (24) was confirmed by immunocytochemistry (Figure 2B/C).

**SSCs proliferation and differentiation**

**Figure 3:**
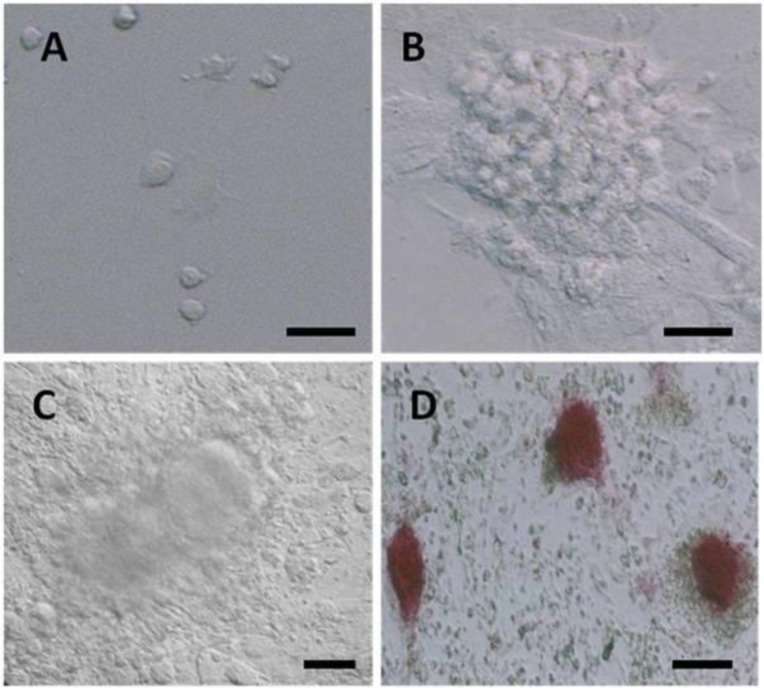
Microscopy morphology of SSCs derived from 4-6 week-old male mice. SSCs were single on 1st day of cell culture (x 400) (A). After 2-3th days of cell culture Small SSCs colonies were formed (x 400) (B), on 7th day of cell culture the size of colonies was increased (x 400) (C) and positive for alkaline phosphatase activity (x 200) (D). Bar 100µm(A, B, C) and 50µm (D).

Isolated Thy1+ SSCs were cultured in the presence or absence of LIF co-culturing Sertoli cells. In presence of LIF, on the first day of culture, SSCs were single and attached to Sertoli cells or gelatin coated dishes (Figure 3A). 

**Figure 4:**
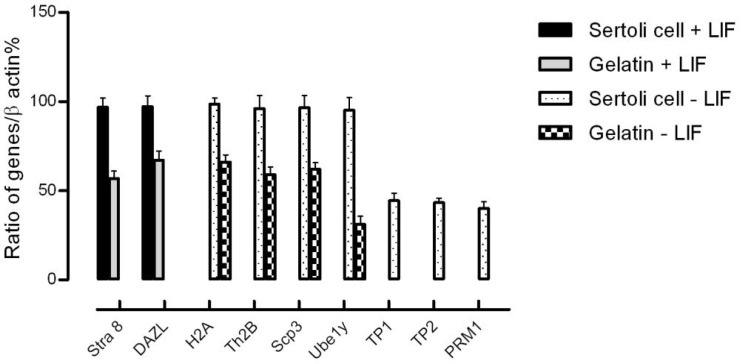
Expression pattern of specific mitotic and meiotic SSC genes after 7 days of cultivation analyzed by quantitative RT-PCR. One way analysis of variance showed that SSCs specific genes (Stimulated by retinoic acid gene: Stra8 and Detected in azoospermia- like: DAZL) expressed only in the presence of LIF, but levels of these genes were higher in Sertoli cell group (p<0.05). The spermatocyte specific genes (H2A, Synaptonemal complex protein 3: Scp3 and Th2b) and one of the spermatid-specific transcripts (Ubiquitin-activating enzyme: Ube1y) expressed in absence of LIF and further rose in Sertoli cell group (p<0.05). The other spermatid-specific transcripts (Transition protein 1: TP1, TP2 and Protamine 1: PRM1) were only detectable in the group co-cultured with Sertoli cells in the absence of LIF (p<0.05

After two days, the majority of round cells aggregated into clumps in which no cytoplasmic bridges could be discerned (Figure 3B), and then colonies were formed on days 2–3 (Figure 3C). These cell colonies were positive for alkaline phosphatase activity (Figure 3D). The cells exhibited expression levels of Stra8 and Dazl, and they lost detectable postmeiotic transcripts, but the expression level of this genes was significantly (p<0.05) higher in the group SSCs co-cultivated with Sertoli cells (Figure 4). Isolated Thy1+ SSCs were cultured in the absence of LIF and co-cultured with gelatin or Sertoli cells. The SSC colonies developed in a limited fashion and lost detectable Stra8 and Dazl expression. In both groups, the cells exhibited expression levels of primary spermatocyte-specific genes, H2A and Scp3 and spermatocyte gene; Th2b. But, Sertoli cells increased significantly the expression level of these genes. It was also detected only one of the spermatid-specific transcripts (Ube1y) in the group SSCs cultured on gelatin. A significant rise of TP1 (p=0.01), TP2 (p=0.005), and PRM1 (p=0.01) cDNA levels were only detectable in the groups co-cultured with Sertoli cells (Figure 4). All genes were expressed in the testis as positive control, but were absent in the liver as the negative control.

**Table I:** Primers sets used for amplification of specific genes

**Genes**	**Primers sequences (5' to 3')**
DAZL	Forward: AAGGCAAAATCATGCCAAAC Reverse: TTCTGCACATCCACGTCATT
Stra8	Forward: CTGTTGGACCAGATGCTGAA Reverse: GCAACAGAGTGGAGGAGGAG
H2A	Forward: GCTGTCACGAAAGTGCAGAAG Reverse: GGTCGAGCGCTTGTTGTAATG
Th2b	Forward: GATGCCGCGAAGAGAGTTACTC Reverse: GGTCGAGCGCTTGTTGTAATGCG
SCP3	Forward: CCGCTGAGCAAACATCTAAAGATG Reverse: GGAGCCTTTTCATCAGCAACAT
Ube1y	Forward: TTTACTCCCGCCAGCTGTAT Reverse: AATGCCCTGATCATGGAGAG
TP1	Forward: GCCGCAAGCTAAAGACTCATGG Reverse: GGGATCGGTAATTGCGACTTG
TP2	Forward: GGCCTCAAAGTCACACCAGTAAC Reverse: TTGCAGGTGAGTGTCGAGAGTGC
PRM1	Forward: ATGGCCAGATACCGATGCTGC Reverse: CAGCATCTTCGCCTCCTCCG
β actin	Forward: TTCTACAATGAGCTGCGTGTGG Reverse: GTGTTGAAGGTCTCAAACATGAT

## Discussion

In mammalian, spermatogenesis completely depends on SSCs. SSCs could self-renew and differentiate in vivo. The low number of SSCs in the testis and lack of specific cell markers are the main problems with these cells (27). Expansion and enrichment of SSCs is a required step for successful transplantation. For enrichment we used MACS and Thy-1 antibody and the purity of this method was evaluated by α6 and β1 integrin (22). Thy-1 is a surface antigen expressed on undifferentiated SSCs and other stem cells (14, 28). Our previous studies have shown that this method was very effective in isolating SSCs from adult and 3-6 day old mice (29, 30). In this study, we used this preparation for adult mice and we could reveal that the Thy-1+ cells had high purity and expressed two specific surface markers for spermatogonial stem cells i.e. α6 and β1-integrin. In this study, by designing the natural niche for SSCs, the number of SSCs was increased and they were differentiated into spermatid-like cells in vitro. This may provide a valuable therapeutic approach to some diseases (31). For isolating adult Sertoli cells, Lectin DSA coated dishes were used, a method has been described earlier by Scarpino *et al* (23). Isolated cells expressed vimentin in their cytoplasm as demonstrated by immunocytochemistry.

SSCs were cultured for 7 days on gelatin and adult Sertoli cells as feeder layer in the presence or absence of LIF. After one week cultivation, gene expression was analyzed ie, Stra8, Dazl, H2A, Scp3, TH2B, Ube1y, TP1, TP2 and PRM1. The data clearly showed that Sertoli cells in the presence of LIF could effectively maintain SSCs in undifferentiated state. The cells expressed Stra8 and Dazl, but they did not show any expression of miotic genes. In the absence of LIF, Sertoli cells promoted differentiation in these cells. They lost detectable Stra8 and Dazl expression and showed miotic gene expression. In this study, it could be also demonstrated that in the absence of LIF, SSCs expressed primary spermatocyte and spermatide specific genes.

It seems, different signaling pathways involved in regulating SSCs fate (32). Testis niche regulates SSCs proliferation and differentiation via paracrine signals. The most important component of this niche is Sertoli cells. Sertoli cells produce different growth factors essential for self-renewal and differentiation of SSCs (6). Previous studies have shown that Sertoli cells were able to successfully maintain SSCs identity (29, 33). As mentioned in the introduction, one of the most important growth factors produced by Sertoli cells is GDNF. In the brain, this factor is secreted by glial cells (34). GDNF is also expressed in the testis by Sertoli cells (12). Over expression of GDNF increases SSCs proliferation (35). This factor acts through different pathways to promote SSCs proliferation. This property of GDNF is mediated by its receptor, GDNF family receptor alpha 1 (GFRα1), and the Ret tyrosine kinase transmembrane protein on SSCs (12) or through members of the Src family of non-receptor tyrosine kinases (36). Another factor that could stimulate SSCs proliferation is LIF (17). This cytokine was added to the basic culture medium for increasing the number of SSCs. LIF is a multifunctional pleiotropic cytokine and has key roles in the regulation of stem cells (37). LIF could maintain embryonic stem cells in the undifferentiated state. LIF receptors are strongly expressed on SSCs, Sertoli cells, testicular macrophages and Leydig cells. It is noteworthy that LIF could not promote gonocyte proliferation for more than one week and the presence of GDNF is required for survival or proliferation of gonocytes (37). By culturing the SSCs on Sertoli cells, GDNF could release to the medium. LIF could suppress apoptosis during the first hours of germ cells isolation and it appears to be unable to inhibit apoptosis for long term culture (37). Therefore, this cytokine was used only for one week.

The data revealed that Sertoli cells in the absence of LIF could induce meiosis in vitro. Tesarik *et al* demonstrated that short term co-culture of germ cells with Sertoli cells can progress spermatogenesis (38). Miryounesi *et al* showed that in the absence of retinoic acid, Sertoli cells could promote embryonic stem cells differentiation (39). Hue *et al* used testis somatic cells for SSCs differentiation. They could differentiate germ cells into spermatid in vitro (40). Sertoli cells produce stem cell factor (SCF), which interacts to its receptor, c-kit, on spermatogonia, spermatocyte and spermatid. SCF/c-kit system not only regulates germ cell apoptosis (41), but also stimulates differentiation in immortalized spermatogonial cell line (42). 

Tajima *et al* showed that other factors produced by Sertoli cells in co-culture system that could promote spermatogonial stem cell differentiation were Insuline like growth factor 1 and Transforming growth factor alpha (43). This agrees consistently with the present findings of improved in vitro differentiation in the presence of Sertoli cells. Taken together, these date indicated that the signals from neighbor cells and the growth factors secreted by these cells could determine the SSCs destiny: maintaining pluripotency or turning to differentiation. By using this property, it would be able to control SSCs fate in vitro for therapeutic strategy to treat male infertility.

## Conclusion

This data demonstrated that Sertoli cells could promote SSCs proliferation and differentiation in vitro, as such providing insight into maturation conditions useful for the treatment of male infertility. 
